# Predicting transcription factor activities from combined analysis of microarray and ChIP data: a partial least squares approach

**DOI:** 10.1186/1742-4682-2-23

**Published:** 2005-06-24

**Authors:** Anne-Laure Boulesteix, Korbinian Strimmer

**Affiliations:** 1 Department of Statistics, University of Munich, Ludwigstr. 33, D-80539 Munich, Germany

## Abstract

**Background:**

The study of the network between transcription factors and their targets is important for understanding the complex regulatory mechanisms in a cell. Unfortunately, with standard microarray experiments it is not possible to measure the transcription factor activities (TFAs) directly, as their own transcription levels are subject to post-translational modifications.

**Results:**

Here we propose a statistical approach based on partial least squares (PLS) regression to *infer *the true TFAs from a combination of mRNA expression and DNA-protein binding measurements. This method is also statistically sound for small samples and allows the detection of functional interactions among the transcription factors via the notion of "meta"-transcription factors. In addition, it enables false positives to be identified in ChIP data and activation and suppression activities to be distinguished.

**Conclusion:**

The proposed method performs very well both for simulated data and for real expression and ChIP data from yeast and *E. Coli *experiments. It overcomes the limitations of previously used approaches to estimating TFAs. The estimated profiles may also serve as input for further studies, such as tests of periodicity or differential regulation. An R package "plsgenomics" implementing the proposed methods is available for download from the CRAN archive.

## Background

The transcription of genes is regulated by DNA binding proteins that attach to specific DNA promoter regions. These proteins are known as transcriptional regulators or transcription factors and recruit chromatin-modifying complexes and the transcription apparatus to initiate RNA synthesis [1,2].

In the last few years, considerable efforts have been made by both experimental and computational biologists to identify transcription factors, their target genes and the sensitivity of the regulation mechanism to changes in environment [3-5]. An important technique for the identification of target genes bound in vivo by known transcription factors is the combination of a modified chromatin immunoprecipitation (ChIP) assay with microarray technology, as proposed by Ren *et al*. [1]. For instance, in the budding yeast *Saccharomyces cerevisiae*, ChIP experiments have been utilized to elucidate the binding interactions between 6270 genes and 113 preselected transcription factors [2]. However, as physical binding of transcription factors is a necessary but not a sufficient condition for transcription initiation, *ChIP data typically suffer from a large proportion of false positives*.

Several attempts have also been made to recover the network structure between transcription factors and their targets using only the gene expression levels of both the transcription factors and the targets, either with [6] or without [7] assuming a subset of putative regulators. Such approaches implicitly assume that the measured gene expression levels of the transcription factors reflect their actual activity. However, owing to various complex post-translational modifications as well as to interactions among transcription factors themselves, *regulator transcription levels are generally inappropriate proxies for transcription factor activities (TFA)*.

In a few recent papers, integrative analysis of gene expression data and ChIP connectivity data has been suggested as a way of overcoming these difficulties [8]. Most prominently, Liao and coworkers have developed the technique of "network component analysis" (NCA) [9,10], a dimension reduction approach to *inferring *the true regulatory activities. In NCA one can also incorporate further a priori qualitative knowledge about gene-transcription factor interactions [11]. Unfortunately, a major drawback of the original NCA method is that for identifiable reasons it imposes very strong restrictions on the network topologies allowed, which renders application of classic NCA difficult in many practical cases. Alter and Golub [12] introduced an approach for integrating ChIP and microarray data using pseudo-inverse projection. Like NCA, this method is based on algebraic matrix decomposition (in this case singular value decomposition). However, this ignores measurement and biological errors present in both connectivity and gene expression data. Kato *et al. *[13] proposed yet another integrative approach consisting of several steps combining sequence data, ChIP data and gene expression data. However, here gene expression is used only to check the coherence of expression profiles of genes with common sequence motifs, and not to estimate transcription factor activities. Finally, Gao *et al*. [14] suggested the "MA-Networker" algorithm, which employs multivariate regression to estimate TFAs and backward variable selection to identify the active transcription factors. Unlike the other approaches, it takes full account of stochastic error. However, for classical regression theory to be valid it is necessary not only that the number of gene targets is much greater than both the number of samples and the number of transcription factors, but also that the transcription factors are independent of each other. The latter condition in particular is clearly not generally satisfied with genome data.

Here, we suggest an alternative statistical framework to tackle the problem of network component and regulator analysis. Our approach centers around multivariate partial least squares (PLS) regression, a well-known analysis tool for high-dimensional data with many continuous response variables that has been widely applied, especially to chemometric data [15-17]. Using PLS we are able not only both to integrate and generalize previous NCA approaches, but also to overcome their respective limitations. In particular, PLS-based network component analysis offers a computationally highly efficient and statistically sound way to infer true TFAs for any given connectivity matrix. In addition, it allows statistical assessment of the available connectivity information, and also the discovery of interactions and natural groupings among regulatory genes (corresponding to "meta"-transcription factors).

## Results

### Network model

Suppose gene expression data for *n *genes and *m *samples (= arrays, tissue types, time points etc.) are collected in a *n *× *m *data matrix . Furthermore, let  denote the so-called connectivity matrix with *n *rows and *p *columns. Each column in  describes the strength of interaction between one of *p *transcription factors and the *n *considered gene targets. The entries of  can either be binary (0–1) or numeric (e.g. ChIP data), with a zero value indicating no physical binding between a transcription factor and a target.

In order to relate expression to connectivity data we consider the linear model



where **A **is a *n *× *m *constant matrix,  is a *p *× *m *matrix of regression coefficients and **E **is a *n *× *m *matrix containing error terms. **A **contains the *m *different offsets, and *may be interpreted as the matrix of the true transcription factor activities (TFAs) of the p transcription factors for each of the m samples*.

It is worth noting that in this setting, unlike in most other gene expression analysis studies, the number of genes *n *is considered as the number of *cases *rather than the number of variables. In the present case the latter corresponds to the number of transcription factors *p *(hence, in general, *p *<*n*).

### NCA and MA-Networker algorithms

The above model linking TFAs both with gene expression of the regulated genes and external connectivity information has been the subject of a series of recent studies.

In the classic network component analysis approach [9,10] the offset matrix **A **is set to zero and the remainder of Eq. 1 is interpreted as a dimension reduction that projects the output layer  with *m *samples on to a "hidden" layer of *p *<*m *transcription factors. In the original NCA algorithm the coefficients  are obtained via a novel matrix decomposition that respects the zero pattern constraint given in the connectivity matrix . Unfortunately, this also imposes rather strict identifiability conditions. As a consequence, classic NCA may only be employed with certain classes of "NCA compatible" [9].

In contrast, the "MA-Networker" algorithm by Gao *et al*. [14] employs standard multiple least-squares regression in conjunction with step-wise variable selection to estimate the true transcription factor activities . This requires that the number of target genes is much larger than both the number of transcription factors and the number of samples. More important, however, is that the step-wise model selection procedure employed is only poorly suited if the regulator genes are themselves interacting with each other. This is a major drawback as it is biologically well-known that transcription factors often work in conjunction with other regulators, and rarely act independently.

### Partial least squares regression

Here we propose to employ the method of partial least squares regression [15] to infer true TFAs and the functional interactions of regulators.

PLS is a well-known analysis tool for high-dimensional data with many continuous response variables that has been widely applied, especially to chemometric data [17]. PLS is particularly suited to the case of non-independent predictors and for small-sample regression settings [16,18-20]. It is computationally highly efficient, it does not necessitate variable selection, and it additionally infers meaningful structural components.

For these reasons PLS is now being adopted as a standard tool for multivariate microarray data analysis, particularly in classification problems [21-24]. We believe that PLS also provides an excellent framework for integrative network analysis, as it combines dimension reduction with regression and variable selection, the two key elements from both the NCA and the MA-Networker approaches.

In a nutshell, the PLS algorithm consists of the following consecutive steps:

1. First, the data matrices  and  are centered to column mean zero, resulting in matrices **X **and **Y**, in order to estimate and to remove the offset **A**. In addition, it is common practice in PLS analysis (and also recommended here) to scale the input matrices to unit variance.

2. Second, using the linear dimension reduction **T **= **XR**, the *p *predictors in **X **are mapped onto *c *≤ rank(**X**) ≤ min(*p*, *n*) latent components in **T **(an *n *× *c *matrix). See the section "SIMPLS algorithm" below for the precise procedure employed in this paper. *The important key idea in PLS is that the weights ***R ***(a p × c matrix) are chosen with the response ***Y ***explicitly taken into account, so that the predictive performance is maximal even for small c.*

3. Next, assuming the model **Y **= **TQ**' + **E**, **Y **is regressed by ordinary least squares against the latent components **T **(also known as X-scores) to obtain the loadings **Q **(a *m *× *c *matrix), i.e. **Q **= **Y**'**T**(**T**'**T**)^-1^.

4. Subsequently, the PLS estimate of the coefficients **B **in **Y **= **XB **+ **E **is computed from estimates of the weight matrix **R **and the Y-loadings **Q **via **B **= **RQ**'.

5. Finally, the coefficients  for the original Eq. 1 are computed by rescaling **B**.

Note that it is step 2 that mostly distinguishes PLS from related bilinear regression approaches such as principal and independent components regression (PCR/ICR) and the pseudo-inverse-based method of Alter and Golub [12]. In the latter approaches the scores **T **are computed solely on the basis of the data matrix **X **without considering the response **Y **[16].

Other quantities often considered in PLS include, e.g., the X-loadings **P **that are obtained by regressing **X **against **T**, i.e. **X **= **TP**' + **F **and **P **= **X**'**T**(**T**'**T**)^-1^.

### SIMPLS algorithm

PLS aims to find latent variables **T **that simultaneously explain both the predictors **X **and the response **Y**. The original ideas motivating the PLS decomposition were entirely heuristic. As a result, a broad variety of different, but in terms of predictive power equivalent, PLS algorithms have emerged – for an overview see e.g. Martens [17].

For the present application to infer true TFAs, we suggest using the SIMPLS ("Statistically Inspired Modification of PLS") algorithm, which has the following appealing properties [18-20]:

• it produces orthogonal, i.e. empirically uncorrelated, latent components;

• it allows for a multivariate response; and

• it optimizes a simple statistical criterion.

A further added advantage of SIMPLS is that it is also one of the most computationally efficient PLS algorithms.

We note that other PLS variants described in the literature have predictive power comparable to SIMPLS. However, these either provide orthogonal loadings rather than orthogonal latent components **T **(Martens' PLS), or they do not elegantly extend from 1-dimensional to *m*-dimensional responses **Y **in terms of their optimized objective function (NIPALS).

In SIMPLS, the latent components **t**_1_, **t**_2_,..., **t**_*c *_of the columns in **T **are inferred by sequentially estimating the column vectors **r**_1_,..., **r**_*c *_of **R **according to the following criterion [20]:

1. **r**_1 _is the unit vector (with |**r**_1_| = 1) maximizing the length |**Y**' **Xr**_1_| of the *m *× 1 covariance vector cov(**Y**, **t**_1_).

2. For all *j *= 2,...,*c*, **r**_*j *_are the unit vectors (with |**r**_*j*_| = 1) maximizing the length |**Y**' **Xr**_*j*_| of the vector cov(**Y**, **t**_*j*_) subject to the orthogonality constraint  for all *i *= 1,...,*j *- 1.

In the actual SIMPLS procedure, the weights **R **and the derived quantities **T **and **Q **are obtained by a Gram-Schmidt-type algorithm [18].

On a practical note, we would like to mention that in many implementations of SIMPLS (e.g. in the "pls.pcr" R package by Ron Wehrens, University of Nijmegen), conventions different from the above are used. In particular, the X-scores T* returned will often be orthonormal (rather than orthogonal) and consequently the weights R* will not have unit norm as in our case. For conversion, define **M **= diag(||,...,||,) and set **T **= T*M^-1^, **R **= R*M^-1^, **Q **= Q*M, and **P **= P*M. This provides orthogonal scores and unit-norm weights as assumed in our description of SIMPLS.

The resulting estimates of the matrices **B**, **T**, and **R **are now straightforward to interpret in terms of transcriptional regulation. **B **(and ) give the inferred activities of the *p *transcription factors in each of the *m *experiments. The inferred latent components **T **describe "meta"-transcription factors that combine related groups of transcription factors. **R **reflects the involvement of each of the *p *regulators in the *c *meta-factors.

### Determining the number of PLS components

A remaining aspect of PLS regression analysis is the optimal choice of the number *c *of latent components. If the maximal value *c*_max _= rank(**X**) is chosen, then PLS becomes equivalent to principal components regression (PCR) with the same number of components, and if additionally *n *>*p *both PLS and PCR turn into ordinary least-squares multiple regression.

Hence, with PLS it is desirable to choose as small a value of *c *as possible without sacrificing too much predictive power. One straightforward statistical procedure to estimate this minimum value *c*_min _is the method of cross-validation, which proceeds as follows (cf. also refs. [25] and [26]):

1. Split the set of *n *genes randomly into 2 sets: a learning set containing 2/3 of the genes and a test set containing the remaining genes.

2. Use the learning set to determine the matrix of regression coefficients **B **for different values *c *= 1, 2,...,*c*_max_.

3. Predict the gene expression of the *n*/3 genes from the test set using **B **with the different values of *c*.

4. Repeat steps 1–3 *K *= 100 times and compute the mean squared prediction error for each *c*.

Subsequently, the value of *c *yielding the smallest mean squared prediction error is selected.

**Figure 1:**
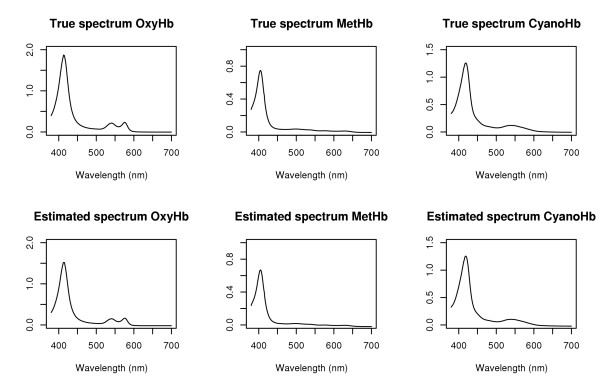
Comparison of true (*top row*) and estimated (*bottom row*) spectra, as obtained by multivariate PLS regression from the validation data set.

**Figure 2:**
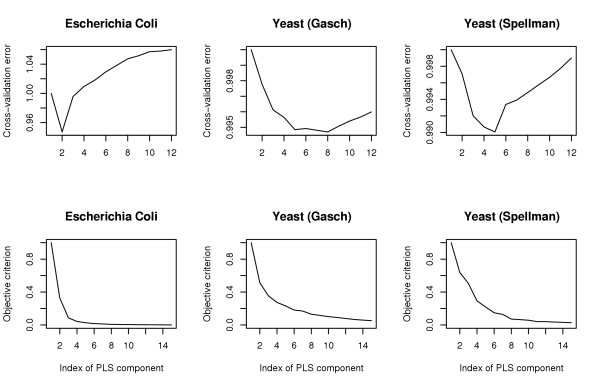
*Top row: *Mean sum of squared prediction error for *E. Coli *and yeast data sets over 100 cross-validation runs. *Bottom row: *maximized objective criterion for each PLS component.

Alternatively, the optimal number of components may also be determined by considering the value of the criterion *Z*_*i *_= |**Y**'**t**_*i*_| for a given latent component **t**_*i*_. If *Z*_*i *_falls below an a priori specified threshold then *c*_min _= *i *is reached.

## Discussion

### Data sets

Next, we illustrate the versatility of the proposed PLS approach to network component analysis by analyzing several real biomolecular data sets.

First, in order to validate the linear regression approach (Eq. 1) we reanalyzed hemoglobin data from Liao *et al. *[9]. Second, we analyzed two different *S. Cerevisiae *gene expression data sets in conjunction with a regulator-target connectivity matrix from the large-scale ChIP experiment of Lee *et al. *[2]. The yeast expression data investigated comprise a time series experiment from Spellman *et al*. [27] and a compilation of yeast stress response experiments from Gasch *et al. *[6,28]. Finally, we analyzed expression and connectivity data for an *E. Coli *regulatory network containing 100 genes and 16 transcription factors from Kao *et al. *[10]. The general characteristics of these four data sets are summarized in Table 1.

**Table 1:** Characteristics of the analyzed data sets.

Data	Reference	n	p	m	*c*_min_
Hemoglobin	[9]	7	3	321	3
*S. cerevisiae*	[27]	3638	113	24	5
*S. cerevisiae*	[6, 28]	1993	113	173	8
*E. coli*	[10]	100	16	23	2

*Abbreviations*: *n*, number of genes; *p*, number of transcription factors; *m*, number of arrays resp. measurements.

The data investigated were preprocessed as follows. The yeast ChIP data set [2] contains protein-DNA interaction data for 6270 genes and 113 transcription factors. It includes missing values that correspond to non-interacting gene-transcription factor pairs. Although ChIP data are essentially continuous, it is common practice to dichotomize them according to the *p*-values into discrete levels of interaction (0 or 1). In this study, we used data obtained at a *p*-value threshold of 0.001, as suggested by Lee *et al. *[2]. However, note that in contrast to the NCA method, dichotomization of the ChIP data is optional in our approach.

The Spellman *et al. *[27] microarray data originally contained the gene expression of 4289 genes at 24 time points during the cell-cycle. From these genes, a subset of 3638 are also contained in the Lee *et al. *[2] ChIP data set. Our analysis is based on these 3638 genes. Similarly, the Gasch expression data set [6,28] contains the expression of 2292 genes for 173 arrays corresponding to different stress conditions (e.g. heat shock, amino acid starvation, nitrogen depletion). Of these 2292 genes, a subset of 1993 overlap with the genes considered in the ChIP data.

The connectivity matrix for the *E. coli *data was compiled mainly by Kao *et al. *[10] from the RegulonDB [11] database. In addition, they incorporated a few corrections using literature data. The temporal *E. coli *expression data for 100 genes across 25 time points was also introduced in Kao *et al. *[10] and is publicly available at .

### Validation of the regression approach

The hemoglobin data used in Liao *et al. *[9] for validation of the classic NCA approach have the advantage that the true coefficients  of the network model in Eq. 1 are known, and therefore can be directly compared with the inferred values.

Reanalyzing these data, it is straightforward to show (see Figure 1) that the true regression coefficients can be recovered exactly by multivariate regression (of which PLS is a special case). According to Liao *et al. *[9], this is also true for classic NCA but not for PCA and ICA interpretations of Eq. 1. This discrepancy can be explained by the fact the neither PCA nor ICA explicitly takes account of the response **Y**, whereas NCA and PLS do.

### PLS components and Y-loadings

Subsequently, we determined the minimum number of PLS components for the yeast and *E. coli *data sets using cross-validation. The results are plotted in Figure 2 (top) after normalization (the mean cross-validation error with one PLS component is set to one). As can be seen from Figure 2, the minimal mean cross-validation error is obtained with 5 PLS components for the Spellman data, 8 PLS components for the Gasch data and 2 PLS components for the *E. coli *data. For comparison, the (normalized) objective criterion |**Y**'**t**_*i*_| of the SIMPLS algorithm is also represented on Figure 2 (bottom) for different numbers of PLS components. These results are in good agreement with the cross-validation error: it increases when PLS components with a low objective criterion are added.

**Figure 3:**
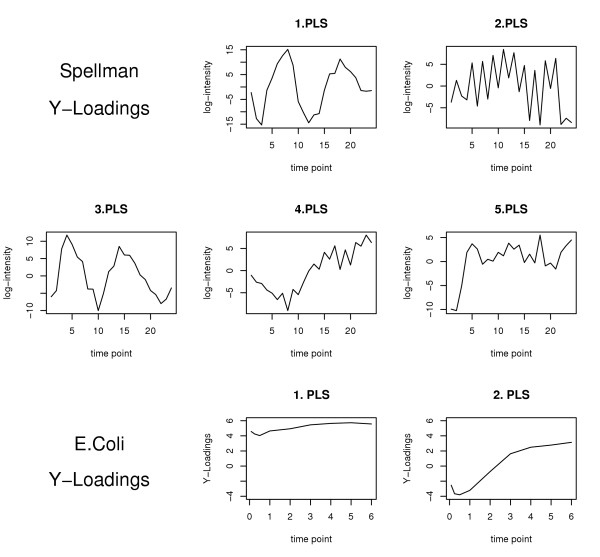
Y-loadings for the *E. Coli *(*top and middle row*) and Spellman (*bottom row*) data sets.

The *Y*-loadings contained in the *m *× *c *matrix **Q **give the projection of the *c *"meta"-transcription factors for each of the *m *experiments. As can be seen from Figure 3 for the Spellman data, both the first and the third meta-factors explain the periodic part of the expression data, but with different phases. The second meta-factor corresponds to small oscillations with very short period, whereas the fourth and fifth meta-factors reflect long-time trends (slow and step-wise increasing, respectively). Using Fisher's *g*-test as proposed in Wichert *et al. *[29], we detected statistically relevant periodicity for the four first meta-factors. In Figure 3, the *Y*-loadings are also represented for the *E. coli *data. Whereas the projection of the first meta-factor is approximately constant over time, the projection of the second meta-factor increases strongly and (almost) uniformly. Thus, in both data sets, the PLS algorithm allows us to extract meta-factors from the data corresponding to distinct latent trends.

For the Gasch data, the *m *experiments do not correspond to different time points but to 13 different stress conditions (see Gasch *et al. *[28] for further details, and Table 2 for the list of conditions). In this case the *Y*-loadings may be interestingly analyzed using Wilcoxon's rank sum test. For each condition *k *and each meta-factor *j*, we tested the *H*_0 _hypothesis that the median of the projection of the *j*-th meta-factor is the same in condition *k *as in all the other conditions ({1,..., *k *- 1, *k *+ 1,..., 13}). In this situation, Wilcoxon's rank sum test is preferable to the well-known two-sample *t*-test, because some of the conditions include only a very small number of experiments. The results obtained with a *p*-value threshold of 0.05 are displayed in Table 2. The entries 1 and 0 correspond to significant and non-significant (FDR adjusted) *p*-values, respectively. As can be seen from Table 2, each PLS component carries a particular pattern of associated significant conditions, indicating that the meta-factors capture a distinct *direction *of the data.

**Table 2:** Significant conditions for the first 8 PLS components of the Gasch yeast data set.

Condition \ PLS Component	1	2	3	4	5	6	7	8	Arrays
Heat shock	0	0	0	0	0	0	0	0	1–9,12–15
Variable temperature shocks	0	0	1	0	1	0	0	0	21–25
Hydrogen peroxide	0	0	0	0	0	1	0	0	36–45
Menadione	0	1	0	0	1	1	0	0	46–54
DTT	0	0	0	0	0	0	0	0	55–69
Diamide	1	1	1	0	0	0	1	1	70–77
Sorbitol osmotic shock	0	0	0	0	0	0	0	0	78–89
Amino acid starvation	0	0	1	1	1	0	1	1	91–95
Nitrogen depletion	0	0	1	0	0	1	1	1	96–105
Diauxic shift	0	0	1	0	0	0	1	0	106–112
Stationary phase	1	1	0	1	1	1	1	0	113–134
Continuous carbon sources	1	0	0	0	0	1	0	1	148–160
Continuous temperatures	1	0	0	0	0	0	1	0	161–173

### Inferred transcription factor activities

One of the main objectives of our PLS-based approach is to estimate the true transcription factor activities (TFAs). Although all the TFAs can be estimated in the same way for the three data sets, we display only the evolution over time of a few interesting TFAs for the two time series data sets (i.e. the Spellman and the *E. coli *data).

**Figure 4:**
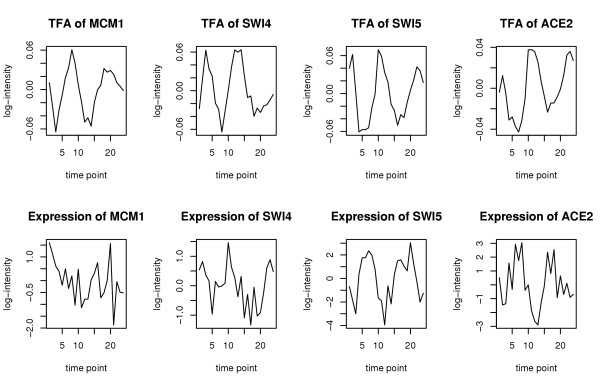
Time profiles of the TFAs (*top row*) of four well-known cell-cycle transcription factors from the Spellman data compared to the respective gene expression measurements (*bottom row*).

The TFAs (top) and expression profiles (bottom) of 4 well-known cell-cycle regulators are depicted in Figure 4 for the Spellman data. The TFAs of MCM1, SWI4, SWI5 and ACE2 show highly periodic patterns, which is consistent with common biological knowledge. In contrast, the *expression *profiles of MCM1 and SWI4 are not periodic (this can be confirmed by Fisher's *g*-test [29]). On the other hand, the expression profiles of SWI5 and ACE2 are periodic, though not with the same phase as the inferred TFAs. This may indicate either an inhibiting or a phase-shift effect of the transcription factors on the regulated genes.

The remainder of the TFAs and the regulated genes were also tested for periodicity using the *g*-test [29]. After FDR adjustment of the *p*-values, we found that 62 of the 113 transcription factors (= 55%) in the Spellman/Lee data have significantly periodic TFAs at the level 0.05. In contrast, only 804 of the 4289 genes (= 19%) exhibit significantly periodic expression profiles.

**Figure 5:**
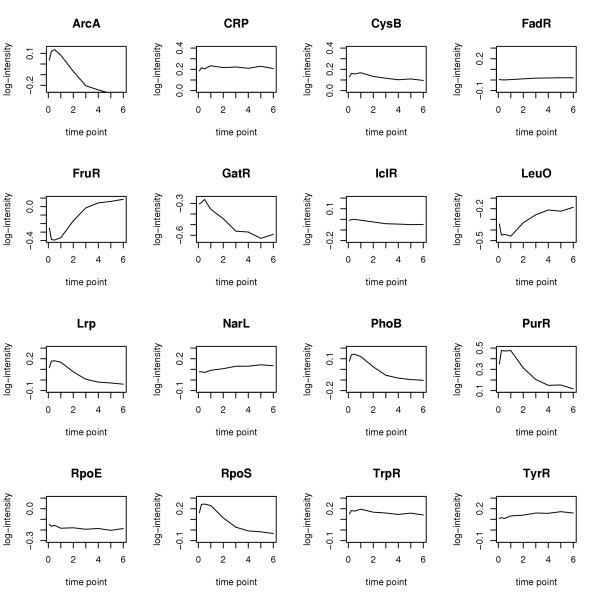
Time profiles of the 16 estimated TFAs (*E. Coli *data).

For the *E. coli *data the time profiles of the estimated TFAs of the 16 transcription factors are represented in Figure 5. The TFAs of ArcA, GatR, Lrp, PhoB, PurR, RpoS decrease over time, those of CRP, CysB, FadR, IcIR, NarL, RpoE, TrpR and TyrR remain approximately constant and those of FruR and LeuO increase strongly. This is consistent with previous results obtained by NCA [10]. We point out, however, that unlike NCA our approach may be applied to any arbitrary network topology, whereas the present *E. coli *network was chosen specifically to meet the NCA compatibility criteria [9].

As can be seen already from the few examples depicted in Figure 4, the TFAs do not always correlate with the respective expression profiles. We tested this for all the transcription factors of which the expression profiles were also included in the data sets. For the Gasch data, we found that only 63 from the 90 available transcription factors exhibit expression profiles that are correlated with TFAs (at the level 0.05 with FDR *p*-value adjustment). For the Spellman time series data, none of the 78 available TFA-expression profile pairs are correlated. These results clearly indicate that methods investigating transcriptional regulation with expression data as their sole basis are likely to miss potentially important regulation activities.

### Gene-regulator coupling factors

Another topic of interest is the identification of false positives in ChIP data. Following Gao *et al. *[14] we investigate this problem using Pearson's correlation test. For each supposed gene-transcription factor pair (according to the dichotomized ChIP data) we test if the inferred TFA is significantly correlated with the expression profile of the regulated gene. For the Gasch data, we find that 73% of the 1495 gene-transcription factor pairs are correct (i.e. the TFA is significantly correlated with the expression profile at the 0.05 level with FDR *p*-value adjustment). The concordance with the ChIP connectivity information is much worse for the Spellman data, where only 32% of the 2535 gene-transcription factor pairs are significantly correlated.

We should like to add as a note of caution that the lack of correlation between TFA and target gene needs to be viewed as specific to the microarray study investigated. Other expression experiments may activate different pathways and thus produce different patterns of correlation in conjunction with the ChIP connectivity information.

## Conclusion

Network component analysis combines microarray data with ChIP data with the aim of enhancing the estimation of regulator activities and of connectivity strengths. In this paper we have presented an approach to NCA based on partial least squares, a computationally efficient statistical regression tool.

Our PLS framework allows several drawbacks, inherent both in the classic NCA methods based on matrix decomposition and in the MA-Networker algorithm, to be overcome. Its simplicity (no iterative step, no variable selection, no stochastic search) and its flexibility (no distributional assumptions, no topological constraints, no conditions on the dimensions) compared to competing approaches make it particularly attractive as an integrative method for analyzing complex regulatory networks. Moreover, the PLS algorithm not only extracts information on gene-regulator and on TFA-expression profile pairs but also identifies coherent meta-factors reflecting the main directions of variation of the data, taking account both of the expression () and the connectivity information ().

Our analysis of biological data shows the versatility of our PLS approach and at the same time dramatically confirms the need for a combined expression-ChIP analysis for inferring regulation. Particularly striking are the sometimes drastic differences between the measured transcription levels and the PLS-inferred transcription activities. According to Segal *et al. *[6], some transcription factors may also not be active in all conditions. Note that this assumption is also automatically taken into account by our approach.

NCA in general, and the present PLS-based variant in particular, may be criticized for relying on a simple linear model - see Buchler *et al. *[30] and Setty *et al. *[31] for counter-examples. Therefore, more elaborate regression approaches such as generalized linear models (GLMs) or generalized additive models (GAMs) may be required to further enhance our current understanding of how best to model the complex structures governing genetic networks.

[*Note added in proof: *See Yang *et al. *[32] for a related study in the sister journal BMC Genomics.]

## Authors' contributions

A.-L.B. performed all the data analysis and simulations. Both authors jointly developed the methodology, wrote the manuscript, and approved of the final version.

## Appendix: Computer program

All algorithms have been implemented in the R language [33]. A corresponding R package "plsgenomics" developed by the authors is available for download from the CRAN archive .

## References

[B1] Ren B, Robert F, Wyrick JJ, Aparicio O, Jennings EG, Simon I, Zeitlinger J, Schreiber J, Hannett N, Kanin E, Volkert TL, Wilson CJ, Bell SP, Young RA (2000). Genome-wide location and function of DNA binding proteins. Science.

[B2] Lee TI, Rinaldi NJ, Robert F, Odom DT, Bar-Joseph Z, Gerber GK, Hannett NM, Harbison CT, Thompson CM, Simon I, Zeitlinger J, Jennings EG, Murray HL, Gordon DB, Ren B, Wyrick JJ, Tagne JB, Volkert TL, Fraenkel E, Gifford DK, Young RA (2002). Transcriptional Regulatory Networks in Saccharomyces cerevisiae. Science.

[B3] Iyer VR, Horak CE, Scafe CS, Botstein D, Snyder M, Brown PO (2001). Genomic binding sites of the yeast cell-cycle transcription factors SBF and MBF. Nature.

[B4] van Steensel B, Delrow J, Bussemaker HJ (2003). Genomewide analysis of Drosophila GAGA factor target genes reveals context-dependent DNA-binding. Proc Natl Acad Sci USA.

[B5] Harbison CT, Gordon DB, Lee TI, Rinaldi NJ, Macisaac KD, Danford TW, Hannett NM, Tagne JB, Reynolds DB, Yoo J, Jennings EG, Zeitlinger J, Pokholok DK, Kellis M, Rolfe PA, Takusagawa KT, Lander ES, Gifford DK, Fraenkel E, Young RA (2004). Transcriptional regulatory code of a eukaryotic genome. Nature.

[B6] Segal E, Shapira M, Regev A, Pe'er D, Botstein D, Koller D, Friedman N (2003). Module networks: identifing regulatory modules and their condition-specific regulators from gene expression data. Nature Genetics.

[B7] Xiong M, Li J, Fang X (2004). Identification of genetic networks. Genetics.

[B8] Li Z, Chan C (2004). Extracting novel information from gene expression data. Trends Biotechnol.

[B9] Liao JC, Boscolo R, Yang YL, Tran LM, Sabatti C, Roychowdhury VP (2003). Network component analysis: reconstruction of regulatory signals in biological systems. Proc Natl Acad Sci USA.

[B10] Kao KC, Yang YL, Boscolo R, Sabatti C, Roychowdhury V, Liao JC (2004). Transcriptome-based determination of multiple transcription regulator activities in Escherichia coli by using network component analysis. Proc Natl Acad Sci USA.

[B11] Salgado H, Santos-Zavaleta A, Gama-Castro S, Millan-Zarate D, Diaz-Peredo E, Sanchez-Solano F, Perez-Rueda E, Bonavides-Martinez C, Collado-Vides J (2001). RegulonDB (version 3.2): transcriptional regulation and operon organization in Escherichia coli K-12. Nucleic Acids Res.

[B12] Alter O, Golub GH (2004). Integrative analysis of genome-scale data by using pseudoinverse projection predicts novel correlation between DNA replication and RNA transcription. Proc Natl Acad Sci USA.

[B13] Kato M, Hata N, Banerjee N, Futcher B, Zhang MQ (2004). Identifying combinatorial regulation of transcription factors and binding motifs. Genome Biology.

[B14] Gao F, Foat BC, Bussemaker HJ (2004). Defining transcriptional networks through integrative modeling of mRNA expression and trasncription factor binding data. BMC Bioinformatics.

[B15] Wold S, Martens H, Wold H, Ruhe A, Kagstrom B (1983). The multivariate calibration method in chemistry solved by the PLS method. Proc Conf Matrix Pencils, Lecture Notes in Mathematics.

[B16] Frank IE, Friedman JH (1993). A statistical view of some chemometrics regression tools. Technometrics.

[B17] Martens H (2001). Reliable and relevant modelling of real world data: a personal account of the development of PLS regression. Chemom Intell Lab Syst.

[B18] de Jong S (1993). SIMPLS: An alternative approach to partial least squares regression. Chemom Intell Lab Syst.

[B19] Braak CJFT, de Jong S (1998). The objective function of partial least squares regression. J Chemometrics.

[B20] de Jong S, Wise BM, Ricker NL (2001). Canonical partial least squares and continuum power regression. J Chemometrics.

[B21] Datta S (2001). Exploring relationships in gene expressions: a partial least squares approach. Gene Expression.

[B22] Nguyen D, Rocke DM (2002). Tumor classification by partial least squares using microarray gene expression data. Bioinformatics.

[B23] Nguyen D, Rocke DM (2002). Partial least squares proportional hazard regression for application to DNA microarray survival data. Bioinformatics.

[B24] Boulesteix AL (2004). PLS dimension reduction for classification with microarray data. SAGMB.

[B25] Dudoit S, Fridlyand J, Speed TP (2002). Comparison of discrimination methods for the classification of tumors using gene expression data. J Amer Statist Assoc.

[B26] Braga-Neto U, Dougherty ER (2004). Is cross-validation valid for small-sample microarray classification?. Bioinformatics.

[B27] Spellman PT, Sherlock G, Zhang MQ, Iyer VR, Anders K, Eisen MB, Brown PO, Botstein D, Futcher B (1998). Comprehensive identification of cell cycle-regulated genes of the yeast Saccharomyces cerevisiae by microarray hybridization. Mol Biol Cell.

[B28] Gasch AP, Spellman PT, Kao CM, Carmel-Harel O, Eisen MB, Storz G, Botstein D, Brown PO (2000). Genomic expression programs in the response of yeast cells to environmental changes. Mol Biol Cell.

[B29] Wichert S, Fokianos K, Strimmer K (2004). Indentifying periodically expressed transcripts in microarray time series data. Bioinformatics.

[B30] Buchler NE, Gerland U, Hwa T (2003). On schemes of combinatorial transcription logic. Proc Natl Acad Sci USA.

[B31] Setty Y, Mayo AE, Surette MG, Alon U (2003). Detailed map of a cis-regulatory input function. Proc Natl Acad Sci USA.

[B32] Yang YL, Suen J, Brynildsen M, Galbraith S, Liao JC (2005). Inferring yeast cell cycle regulators and interactions using transcription factor activities. BMC Genomics.

[B33] R Development Core Team (2004). R: A language and environment for statistical computing.

